# YpeB stability affects germination possibly through delaying SleB activity in *Bacillus subtilis*

**DOI:** 10.1128/jb.00145-26

**Published:** 2026-06-22

**Authors:** Marcel Shams-Eddin, Alexandra Pinkham, Naomi Williams, David L. Popham

**Affiliations:** 1 Department of Biological Sciences, Virginia Tech 1757, Blacksburg, Virginia, USA; 2 Center for Emerging, Zoonotic and Arthropod-borne Pathogens, Virginia Tech 1757, Blacksburg, Virginia, USA; The Ohio State University, Columbus, Ohio, USA

**Keywords:** spore, germination, cortex, lytic, protease, peptidoglycan

## Abstract

Bacterial endospores formed by Bacilli and Clostridia can persist for decades and survive a wide range of killing mechanisms but can rapidly germinate in permissive environments. SleB and CwlJ are partially redundant germination-specific lytic enzymes that degrade *Bacillus* spore cortex peptidoglycan, contributing to the transition to the vegetative state. SleB interacts with YpeB, which may hold it inactive until germination starts. YpeB is rapidly proteolyzed during germination, which may release SleB to degrade the cortex. HtrC cleaves YpeB, but YpeB is still degraded in the absence of HtrC, suggesting the contribution of other proteases that are associated with the inner membrane. We hypothesize that at least one additional protease serves a YpeB degradation role. A mutant strain lacking five spore-associated proteases had significantly delayed spore germination and YpeB degradation when compared to the wild type. It is hypothesized that delayed YpeB degradation might cause SleB to become active later during germination. To further characterize the role of YpeB stability on SleB activity, cortex muropeptides were analyzed using HPLC and mass spectrometry. A decrease in two SleB-associated muropeptides was seen in the protease mutant strain after 30 and 60 min of germination when compared to the WT, which might be the consequence of delayed SleB activity. Identifying protein-protein interactions during germination could guide manipulation of spores either to increase the germination efficiency, rendering the spores more susceptible to killing, or to prevent germination, making the spores inefficient in causing human disease.

**IMPORTANCE:**

Bacterial endospores are agents of some human and animal diseases and of food spoilage. While dormant spores can persist for decades and survive a wide range of killing mechanisms, they must germinate to exert negative effects. Previous studies have indicated that membrane-associated proteases may play a role in a key germination process of cell wall degradation. A *Bacillus subtilis* strain lacking multiple spore proteases was studied and found to exhibit delays in some germination processes. An understanding of, and ability to manipulate, important steps in spore germination can contribute to the development of improved methods for spore inhibition and inactivation.

## INTRODUCTION

Bacterial endospores produced by Bacilli and Clostridia are the most resistant forms of life on earth (1). Resistance properties vary among species, but the majority of spores are resistant to heat, cold, UV irradiation, desiccation, and chemicals including disinfectants and antibiotics (2). Spores are formed in response to nutrient deprivation and can persist in stressful environments and harsh conditions in a dormant stage for decades (3). In contrast to vegetative cells, spores possess a modified structure and internal composition that contribute to their resistance (3). These factors allow spores released by pathogenic *Bacillus* and *Clostridia* species to pose major threats to humans and animals (2, 4).

The spore structure is made up of a multilayered envelope and an inner spore core. The envelope of most spore-formers includes an exosporium, spore coat, outer membrane, cortex, germ cell wall, and inner membrane, but *Bacillus subtilis* spores do not have an exosporium (1). Each of these structures plays roles in protecting the core against damage (5). The spore peptidoglycan (PG) consists of a thick outer cortex layer and a thin inner germ cell wall. Compared to vegetative cell peptidoglycan, the cortex layer contains several structural modifications. The major modification includes the transformation of 50% of the N-acetylmuramic acid (NAM) residues into muramic-δ-lactam (MAL) (6). The presence of MAL renders the cortex susceptible to Germination-Specific Lytic Enzymes (GSLEs) as opposed to the thin germ cell wall that is not degraded in the germination process (7). The inner spore membrane is a major area of interest as it contains most of the proteins required for germination and has a unique physical state with immobile lipids (8). The spore core contains essential material for vegetative cell survival including the genetic material, enzymes, ribosomes, and energy sources. It is highly dehydrated and contains a very high concentration of calcium dipicolinic acid (Ca^2+^-DPA) (9). Spores can rapidly germinate upon exposure to germinants (10), which are often nutrients such as amino acids and sugars, but in varied species can include other environmental signals such as K^+^ ions, bile acids, or Ca^2+^-DPA. Upon exposure to germinants, *B. subtilis* spores release monovalent ions and the large deposit of Ca^2+^-DPA and take up water (10). This process is followed by cortex PG degradation by two partially redundant GSLEs, SleB and CwlJ (11, 12).

GSLEs are produced during sporulation and held stable and inactive in the dormant spore until germination is triggered (13). CwlJ is produced in the mother cell and is associated with the spore coats, while SleB is produced within the developing forespore and is translocated across the inner spore membrane (12, 14, 15). CwlJ is activated when exposed to high concentrations of Ca^2+^-DPA (16), while the mechanisms by which SleB is held inactive in dormant spores and activated during germination are still unclear. SleB is a lytic transglycosylase that can cleave the β-1,4-glycosidic bond between NAM and N-acetylglucosamine in PG, like lysozyme, but SleB creates a new bond between the C-6 hydroxyl group and the C-1 of the same NAM residue, forming a 1,6-anhydromuramic acid residue (17–20).

SleB and its partner protein YpeB are expressed from a conserved operon. YpeB is expressed with a transmembrane domain that should remain in the inner spore membrane with the majority of the protein outside the membrane. SleB is present in its mature form in the dormant spore as it is translocated across the inner membrane, and its signal sequence is removed during sporulation (15, 21, 22). Both SleB and YpeB are required for stable incorporation of each other into the spore inner membrane (22–24). In the absence of one of the proteins, the partner protein is degraded rapidly after translation (22, 23).

*Bacillus anthracis* YpeB is cleaved rapidly during germination by the serine-type endopeptidase HtrC, producing stable 28 and 31.7 kDa fragments (25). A Δ*htrC* mutation failed to fully stabilize YpeB and resulted in altered YpeB proteolysis, generating various non-specific products. Similar HtrC effects on YpeB degradation during germination were observed in *B. subtilis* (25).

Proteomic analysis of the *B. subtilis* inner spore membrane revealed the presence of five additional putative proteases (YugP, YtmA, YmfF, YmfH, and MlpA [YmxG]) (26). It is hypothesized that YpeB is involved in holding SleB inactive on the inner spore membrane until germination starts, and YpeB degradation early during germination helps release SleB to degrade the cortex peptidoglycan, leading to spore rehydration and outgrowth. The goal of the current study is to further characterize the effects of multiple proteases on YpeB stability and degradation, and subsequent effects on SleB activity during *B. subtilis* spore germination.

## MATERIALS AND METHODS

### Strain construction

*B. subtilis* strains are listed in Table 1. All strains used for data generation were derivatives of PS832. The primers used for plasmid construction and for loci verification using PCR are listed in Table 2. Protease-encoding gene mutations were from strains acquired from the Bacillus Genetic Stock Center (BGSC), which carried single-gene deletions, antibiotic resistance gene insertions, and flanking LoxP sites (27). The Cre-recombinase-carrying plasmid pDR244, from BGSC ECE274, was used to remove antibiotic resistance cassettes from the gene deletions (28). To tag the C-terminus of YpeB with Myc, the last 373 bp of *ypeB* were PCR amplified from the *B. subtilis* chromosome. The PCR product was inserted into the vector pMUTIN-Myc (29) using restriction enzymes KpnI and ClaI. After ligation, the circularized vector was electroporated into *Escherichia coli* to generate pDPV1051, and the gene fusion was verified by DNA sequencing. This plasmid was transformed into *B. subtilis* to recombine with the *ypeB* native locus, generating a full-length *ypeB-Myc*. This construct was inserted into various strains (Table 1) for YpeB-Myc stability assays.

**TABLE 1:** Bacterial strains and plasmids

*B. subtilis* strain	Genotype^*a*^	Construction^*b*^	Source or reference
PS832	WT		Peter Setlow (30)
BKK16710	Δ*mlpA::Kan^r^*		BGSC (27)
BKK16845	Δ*ymfF::Kan^r^*		BGSC (27)
BKK16860	Δ*ymfH::Kan^r^*		BGSC (27)
BKK35080	Δ*ytmA::Kan^r^*		BGSC (27)
BKK31310	Δ*yugP::Kan^r^*		BGSC (27)
BKK22920	Δ*ypeB::Kan^r^*		BGSC (27)
BKK22930	Δ*sleB::Kan^r^*		BGSC (27)
DPVB893	Δ*mlpA::Kan^r^*	BKK16170 → PS832	This study
DPVB894	Δ*ymfF::Kan^r^*	BKK16845 → PS832	This study
DPVB895	Δ*ymfH::Kan^r^*	BKK16860 → PS832	This study
DPVB896	Δ*ytmA::Kan^r^*	BKK30580 → PS832	This study
DPVB152	Δ*sleB::Spec^r^*	FB112 → PS832	(16)
DPVB897	Δ*yugP::Kan^r^*	BKK31310 → PS832	This study
DPVB903	Δ*mlpA::Kan^r^,* Δ*htrC::MLS^r^*	DPVB668 → DPVB889	This study
DPVB905	Δ*ymfF::Kan^r^,* Δ*htrC::MLS^r^*	DPVB668 → DPVB904	This study
DPVB906	Δ*ymfH::Kan^r^,* Δ*htrC::MLS^r^*	DPVB668 → DPVB900	This study
DPVB907	Δ*ytmA::Kan^r^,* Δ*htrC::MLS^r^*	DPVB668 → DPVB901	This study
DPVB908	Δ*yugP::Kan^r^,* Δ*htrC::MLS^r^*	DPVB668 → DPVB902	This study
DPVB1108	Δ*htrC::MLS^r^,* Δ*ytmA,* Δ*yugP*	pDR244 → DPVB1107	This study
DPVB1126	Δ*htrC,* Δ*yugP,* Δ*ytmA,* Δ*mlpA*	pDR244 → DPVB1125	This study
DPVB1127	Δ*htrC::MLS^r^*	DPVB1112 → PS832	This study
DPVB1147	Δ*yugP*, Δ*ytmA,* Δ*htrC,* Δ*mlpA,* Δ*ymfF*	pDR244 → DPVB1145	This study
DPVB1152	Δ*yugP*, Δ*ytmA,* Δ*htrC,* Δ*mlpA,* Δ*ymfF,* Δ*cwlJ::Tet^r^*	DPVB145 → DPVB1147	This study
DPVB1165	Δ*yugP*, Δ*ytmA,* Δ*htrC,* Δ*mlpA,* Δ*ymfF, ypeB-Myc:MLS^r^*	pMUTIN-*ypeB-Myc* → DPVB1147	This study
DPVB1166	Δ*yugP*, Δ*ytmA,* Δ*htrC,* Δ*mlpA,* Δ*ymfF,* Δ*cwlJ::Tet^r^, ypeB-Myc:MLS^r^*	pMUTIN-*ypeB-Myc* → DPVB1152	This study
DPVB1167	*ypeB-Myc:MLS^r^*	pMUTIN-*ypeB-Myc* → PS832	This study
DPVB1169	Δ*htrC,* Δ*yugP,* Δ*mlpA,* Δ*ymfH::MLS^r^*	DPVB886 → DPVB1170	This study
DPVB1170	Δ*htrC,* Δ*yugP,* Δ*mlpA::Kan^r^*	DPVB893 → DPVB1148	This study
DPVB1171	Δ*htrC,* Δ*yugP,* Δ*ymfF::Kan^r^*	DPVB894 → DPVB1148	This study
DPVB1172	Δ*htrC,* Δ*yugP,* Δ*ymfH::Kan^r^*	DPVB895 → DPVB1148	This study
DPVB1175	Δ*htrC,* Δ*yugP,* Δ*ymfF::Kan^r^,* Δ*mlpA::MLS^r^*	DPVB884 → DPVB1171	This study
DPVB1176	Δ*htrC,* Δ*yugP,* Δ*ymfH::Kan^r^,* Δ*ytmA::MLS^r^*	DPVB887 → DPVB1172	This study

^*a*^ Tet^r^, tetracycline resistance; *MLS^r^*, macrolide-lincosamide-streptogramin B resistance; Kan^r^, kanamycin resistance; Spec^r^, spectinomycin resistance.

^*b*^ Strains were constructed by natural transformation. The designation preceding the arrow is the plasmid or source of the donor DNA, while the designation following the arrow is the recipient strain.

**TABLE 2:** Primer sequences

Primer name	Sequence (5′ to 3′)	Primer function
796	TACGGCGGAAAGTCTTGAAGC	PCR amplification of *mlpA*
797	TTGATGTCTACAATTTCCTTTCCCG	PCR amplification of *mlpA*
792	GCATGCGGAGGTTATTGTTTC	PCR amplification of *ymfF*
793	AAAGGGACAAACCGGTTATCTATC	PCR amplification of *ymfF*
790	TGAGTTATATCAGCAGGCCG	PCR amplification of *ymfH*
791	AGCAGCAGATTGTATCCTCTAGC	PCR amplification of *ymfH*
798	CCATCTGTTCACATAGACAACCTCC	PCR amplification of *ytmA*
799	ACTTTGTTTTGCGTACATCCATTGG	PCR amplification of *ytmA*
794	CCTTTTATTTGTTTTCAGCAGGCC	PCR amplification of *yugP*
795	GAACCTCTGATTTTGACGATCGC	PCR amplification of *yugP*
1010	GGCCATGGTACCCGGTGTATTTTCATATGTTCCTG	PCR amplification of *ypeB* with KpnI for Myc tagging
1011	GGCCATATCGATTAGGTCTTTATATATAGGTTCTGC	PCR amplification of *ypeB* with ClaI for Myc tagging

### Spore preparation

To prepare spores, *B. subtilis* was grown in liquid 2xSG medium (31) with shaking for 3–4 days at 37°C. Spores were harvested by centrifugation at 4°C and 4,100 × *g* for 15 min; the supernatant was discarded, and the spores were resuspended and washed three times with cold sterile deionized water. Spores were then stored at 4°C and washed once per day for several days until the spores appeared >95% free of other cell debris.

### Spore germination assays

For germination rate, 0.5 OD_600nm_ units of spores were heat-activated at 75°C for 30 min and quenched on ice for 5 min. Spores were then incubated at 37°C with periodic shaking in 25 mM HEPES at a starting OD of 0.2. 10 mM L-valine was used to stimulate germination, and the change in OD was recorded. For outgrowth rate, spores were inoculated to an OD_600nm_ of 0.2 at 37°C with periodic shaking in 2xYT medium (16 g/L tryptone, 10 g/L yeast extract, and 5 g/L NaCl) that was used to stimulate germination and provide the nutrients for spores to outgrow; the change in OD was recorded. For germination and outgrowth efficiency, spores were tested for colony formation. Spores were serially diluted in sterile H_2_O from a starting OD_600nm_ of 0.2 and plated on 2xYT media overnight at 37°C prior to colony counting.

### Spore sample preparation and Western blotting

Dormant spores (7.5 OD units) were prepared in 10 mM Tris-HCl (pH 7.0), and either pelleted, frozen, and lyophilized for dormant samples or used for germination. For germinating spores, samples were heat-activated at 75°C for 30 min. Samples were then cooled on ice for 10 min. Spores were germinated with 10 mM L-valine as described above, except 10 µg/mL chloramphenicol was added to inhibit protein synthesis. Samples were collected 5, 30, and 60 min post-germination, centrifuged at 15,800 × *g* for 30 s, and cell pellets were frozen and lyophilized. Dried samples were broken using 0.1 mm glass beads in a dental amalgamator at 4,200 rpm for 20 pulses of 30 s each. Samples were left to rest for 30 s on ice between pulses. Following breakage, samples were extracted with SDS-PAGE loading buffer (62.5 mM Tris-HCl, pH 6.8, 2% SDS, 10% glycerol, 5% β-mercaptoethanol, and 0.05% bromophenol blue) and heated at 100°C for 10 min. Samples were centrifuged at 15,800 × *g* for 1 min, and the supernatant was stored at −80°C prior to analysis by Western blotting.

Samples were separated on a stain-free gel (Bio-Rad) and transferred onto PVDF membranes. Total protein normalization was performed using the stain-free blot technology (Bio-Rad). After brief exposure to UV light, stain-free fluorochromes bind to the protein molecules in the gel, allowing for protein visualization on the membrane after transfer. Total protein per lane was calculated from the stain-free blot, and each sample was normalized to the total protein in the dormant sample. Anti-Myc monoclonal antibodies (Merck) and anti-SleB polyclonal antibodies were used at dilutions of 1:3,000 and 1:1,000, respectively. Horseradish peroxidase (HRP)-conjugated secondary goat anti-mouse antibodies (Merck) and HRP-conjugated secondary goat anti-rabbit antibodies (Bio-Rad) were used at a dilution of 1:1,000 and 1:200,000, respectively. Antibody detection was carried out using chemiluminescent substrates (Clarity Max Western ECL substrate; Bio-Rad). Bio-Rad Image Lab 6.1 software was used to perform data analysis and normalization for the quantitative blots. Western blot band intensities were normalized to the total protein present in each lane (32). At least three biological replicates were performed, and the adjusted band volume intensities were then plotted as mean ± SD. Independent samples *t*-test was performed, and a *P*-value <0.05 was considered significant.

### Spore peptidoglycan structure analysis

For germination, spores were washed, and 30 OD units were resuspended in 10 mM Tris-HCl at pH 7 and heat-activated at 75°C for 30 min. Spores were then quenched on ice for 10 min before being germinated using 10 mM L-Val for 30 or 60 min. Germinated and dormant spore samples were centrifuged at 15,000 × *g* for 45 s, and the pellet was resuspended in 1 mL 50 mM Tris-HCl at pH 7.5, 1% SDS, 50 mM DTT, and placed in a boiling water bath for 20 min. Spores were washed with sterile dH_2_0 until the supernatant was SDS-free. Samples were treated with 0.1 mg/mL trypsin in 20 mM Tris-HCl at pH 8.0, 10 mM CaCl_2_, at 37°C, overnight. Samples were then heated for 15 min at 100°C with 1% SDS for trypsin inactivation. Samples were centrifuged for 45 s at 15,000 × *g* and washed with sterile dH_2_0 until SDS was undetectable. The pellet was then digested overnight with 125 units of Mutanolysin (Sigma-Aldrich) in 125 µL 12.5 mM NaPO_4_ pH 5.5 at 37°C. Samples were centrifuged at 13,000 × *g* for 15 min, and the supernatant containing the muropeptides was collected, lyophilized, and stored at −20°C. Just prior to loading on HPLC, samples were resuspended in 100 µL 0.25 M Na_2_B_4_O_7_ pH 9, and pH was adjusted to 9 using 1 N NaOH, if necessary. Terminal sugars were reduced for 5 min by adding 25 µL of freshly prepared 25 mg/mL NaBH_4_ in 0.25 M Na_2_B_4_O_7_ at pH 9. The reaction was terminated by adding 3.5 µL of 15 M H_3_PO_4_ and vortexing thoroughly. Sample pH was adjusted to 2, and insoluble material was pelleted by centrifugation at 13,000 × *g* for 5 min before the supernatant was loaded onto the HPLC. Muropeptides were separated using a methanol gradient as previously described (33), and muropeptides were detected by absorbance at 206 nm.

### Mass spectrometry analysis of the muropeptides

Muropeptide samples prepared for HPLC analysis were analyzed by LC-MS for structural confirmation and relative quantification. Analyses were performed on a Shimadzu LCMS9030 QToF mass spectrometer coupled with LC-40B X 3 UPLC system as described in reference 34. Separation was achieved on a Waters BEH C18 column (2.1 × 100 mm, 1.7 µm) using 0.1% formic acid in water (solvent A) and 0.1% formic acid in methanol (solvent B) at 0.3 mL/min. The gradient was 1% B for 3 min, increased linearly to 8% B at 12 min, 20% B at 24 min, and 95% B at 25 min, held for 4 min, followed by re-equilibration to starting conditions. Injection volume was 2 µL. The first 1.25 min of eluent was diverted to waste. Mass spectra were acquired in positive electrospray ionization mode over 300 to 2,000 m/z. For statistical comparisons, data were collected in MS mode only. Muropeptides were identified based on the monoisotopic masses of the protonated (M + H) and/or sodium-adduct (M + Na) ions and, when necessary, confirmed by MS/MS fragmentation patterns. Extracted-ion chromatograms were generated for dormant spore peptidoglycan peaks (PG peaks 1, 2, and 3) and SleB-dependent germination peaks (G5 and G6). Peak areas were integrated and expressed as relative abundance representing ion counts with respect to the total detected muropeptide signal. Values were normalized to PG peak 1, which served as the internal control.

## RESULTS

### Germination and outgrowth kinetics of strains lacking protease-encoding genes

**Fig 1:**
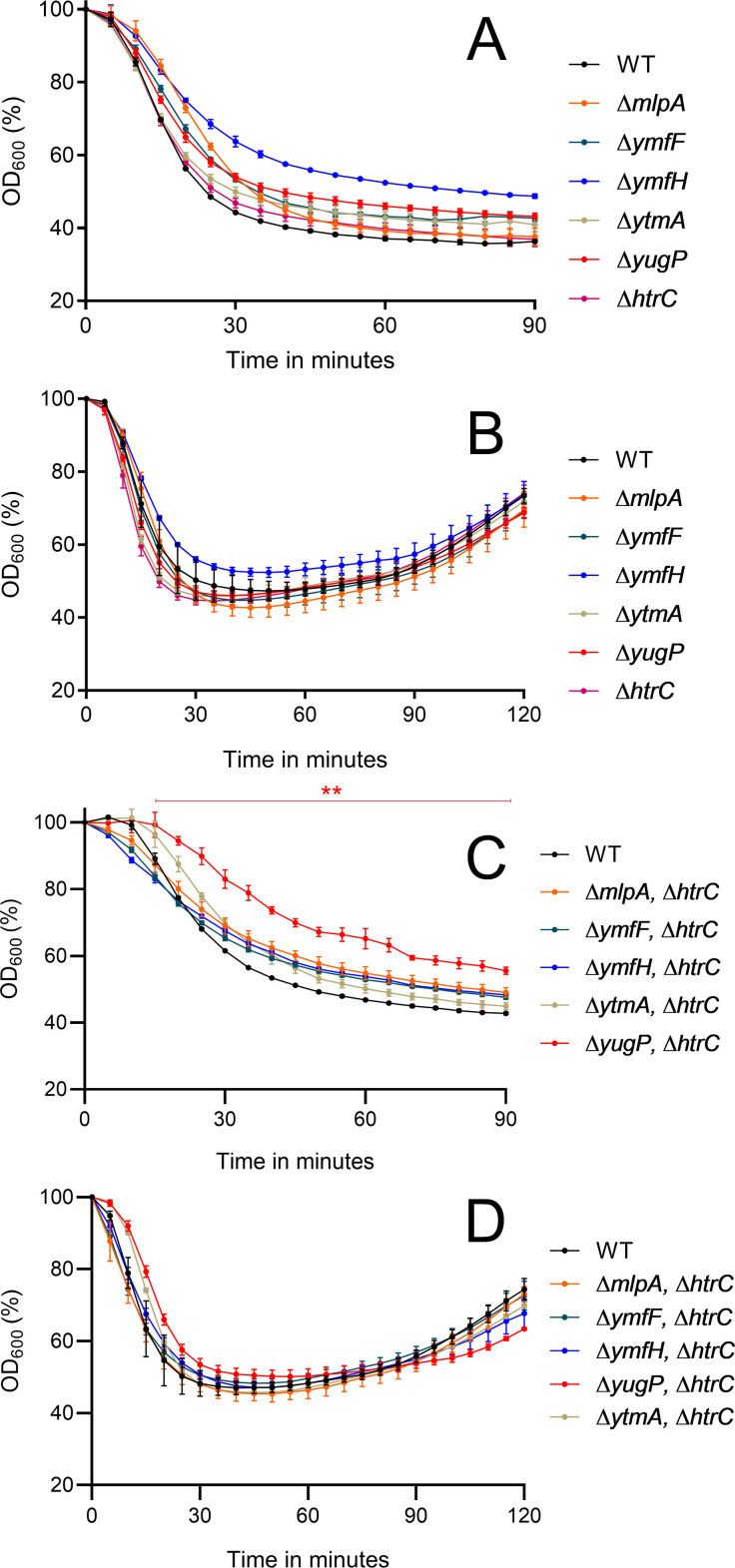
Effects of protease-encoding gene deletions on rates of germination and outgrowth of *B. subtilis* spores. Spores were prepared and purified as described in Materials and Methods. For germination (**A and C**), spores were heat-activated, chilled, and exposed to 10 mM L-valine, and OD was tracked and plotted as a percentage of initial OD. For outgrowth (**B and D**), spores were germinated in 2xYT. Plotted points are means ± SD of five biological replicates. *P*-value ≤0.05 was considered significant (** for Δ*yugP*, Δ*htrC* strain). (**A and B**) Germination kinetics and outgrowth of strains lacking single protease genes, respectively. (**C and D**) Germination kinetics and outgrowth of strains lacking *htrC* and one of the five putative protease genes, respectively.

Proteomic analyses previously identified five putative membrane-associated proteases associated with the inner spore membrane of *Bacillus subtilis*, YugP, YtmA, YmfF, YmfH, and MlpA (26). YugP, YmfF, YmfH, and MlpA are putative metalloproteases, and YtmA is a predicted serine-type endopeptidase (35). Because YpeB is proteolyzed early during germination and this degradation may contribute to the activation of the cortex lytic enzyme SleB, we hypothesized that one or more of these proteases play a role in YpeB degradation and affect germination. Strains lacking single and multiple proteases were constructed using gene deletions from the Koo collection (27) and the Cre-Lox recombinase to remove antibiotic resistance cassette insertions. Spores were prepared, purified, and tested for their germination rates. All strains exhibited sporulation rates and efficiencies similar to the wild type when observed using phase-contrast microscopy 24 h after inoculation, and all spores behaved similarly to those of the wild type during purification. Spores were heat-activated at 75°C and germinated at 37°C using 10 mM L-Val. Single protease deletions did not cause significant changes in the rates of germination and outgrowth of spores (Fig. 1A and B). It was previously found that a deletion of *htrC* did not slow germination (25), and this was also verified in this study. Double deletion strains lacking HtrC and one additional protease were tested. Among the double deletion strains, only that lacking *yugP* and *htrC* significantly delayed germination, but all of the double deletions did not affect spore outgrowth (Fig. 1C and D). Various triple and quadruple mutant strains lacking YmfH caused a significant defect in germination (Fig. S1A). Triple and quadruple mutants did not affect the outgrowth of the spores (Fig. S1B).

The germination efficiency of protease mutant spores was tested by colony formation on rich medium (Table 3). Spores lacking both CLEs (SleB and CwlJ) cannot complete germination (12, 36), and this causes a 10^4^-fold decrease in colony formation relative to the wild-type strain. Protease mutant spores did not exhibit any deficiency in plating efficiency when compared to WT.

**TABLE 3:** Germination efficiency of various protease mutants^*a*^

Strain	CFU/OD unit
WT	(3.1 ± 0.5) × 10^7^
Δ*htrC*, Δ*yugP*, Δ*mlpA*, Δ*ytmA*, Δ*ymfF*	(5.1 ± 0.9) × 10^7^
Δ*htrC*, Δ*yugP*, Δ*mlpA*, Δ*ytmA*, Δ*ymfF*, Δ*cwlJ::Tet*	(9.0 ± 0.9) × 10^7^
*htrC::MLS^r^*, Δ*ytmA*, Δ*yugP*	(3.5 ± 0.3) × 10^7^
Δ*htrC*, Δ*yugP*, Δ*ytmA*, Δ*mlpA*	(2.3 ± 0.4) × 10^7^
Δ*htrC*, Δ*yugP*, Δ*mlpA*, Δ*ymfH::MLS^r^*	(2.7 ± 0.4) × 10^7^
Δ*htrC*, Δ*yugP*, Δ*mlpA::Kan^r^*	(2.1 ± 0.3) × 10^7^
Δ*htrC*, Δ*yugP*, Δ*ymfF::Kan^r^*	(2.2 ± 0.7) × 10^7^
Δ*htrC*, Δ*yugP*, Δ*ymfH::Kan^r^*	(2.0 ± 0.1) × 10^7^
Δ*htrC*, Δ*yugP*, Δ*ymfF::Kan^r^*, Δ*mlpA::MLS^r^*	(2.6 ± 0.4) × 10^7^
Δ*htrC*, Δ*yugP*, Δ*ymfH::Kan^r^*, Δ*ytmA::MLS^r^*	(2.9 ± 0.2) × 10^7^
Δ*cwlJ::Tet^r^, sleB::Spec^r^*	(2.1 ± 0.7) × 10^3^***
Δ*cwlJ::Tet^r^*	(4.2 ± 1.0) × 10^7^
Δ*sleB::Spec^r^*	(5.9 ± 2.0) × 10^7^
Δ*cwlJ::Tet^r^, sleB::Spec^r^*	(2.1 ± 0.7) × 10^3^***
Δ*cwlJ::Tet^r^*, Δ*ypeB::MLS^r^*	(3.0 ± 1.4) × 10^3^***
Δ*cwlJ::Tet^r^, ypeB-Myc::MLS^r^*	(2.4 ± 0.3) × 10^7^

^*a*^ *P*-value less than 0.05 was considered significant (***). Values are mean ± SD from three biological replicates.

### Germination and outgrowth kinetics of Δ5 strain lacking *mlpA, ymfF, ytmA, yugP,* and *htrC*

**Fig 2:**
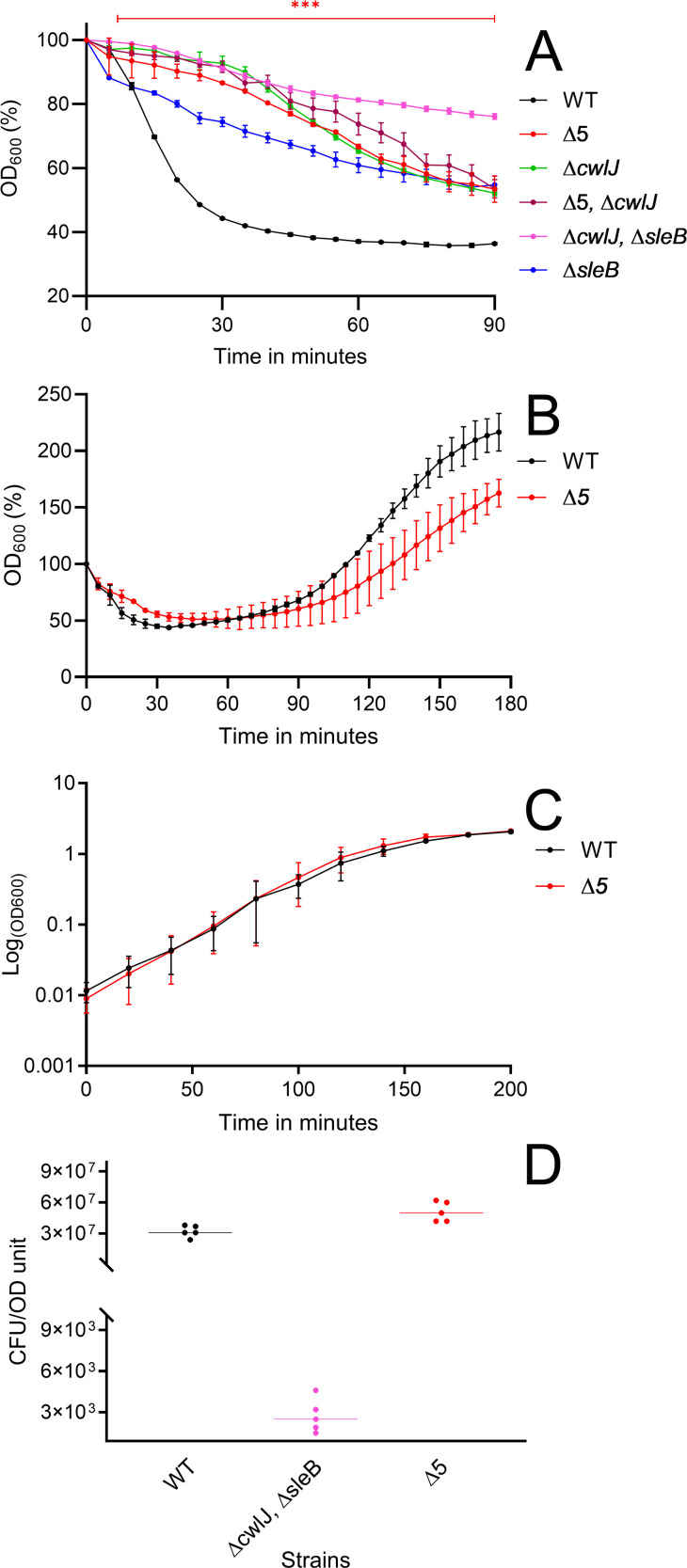
Effects of deletion of five protease-encoding genes on rates of germination and outgrowth of *B. subtilis* spores. Spores were prepared and purified as described in Materials and Methods. For germination (**A**), spores were heat-activated, chilled, and exposed to 10 mM L-valine, and OD was tracked and plotted as a percentage of initial OD. For outgrowth (**B**), spores were germinated in 2xYT. Plotted points are means ± SD of five biological replicates. *P*-value ≤0.05 was considered significant (*** for Δ5 strain). (**A and B**) Germination kinetics and outgrowth of the Δ5 strain, respectively. (**C**) Growth of *B. subtilis* vegetative cells in 2xYT media. (**D**) Germination (plating) efficiency of the spores.

While WT spores rapidly lost 52% of their OD, the Δ5 spores lost only 14% after 30 min of germination (Fig. 2A). A delay in germination was also seen in the first 30 min of the outgrowth assay (Fig. 2B), but was less extreme. As 2xYT medium contains nutrients that induce germination via both the GerA and GerB pathways, this may reduce the germination delay (37). This change in the germination kinetics caused a corresponding delay in outgrowth of the Δ5 spores, but spores were able to outgrow and gain OD after 90 min, when compared to the WT spores that started gaining OD after 40 min (Fig. 2B; Table 3). Vegetative cell growth of the Δ5 strain was similar to that of the WT, with both strains having a doubling time of 20 min (Fig. 2C).

The partial redundancy of CwlJ and SleB can be observed in the germination kinetics of a Δ*cwlJ* mutant versus a Δ*cwlJ* Δ*sleB* double mutant (Fig. 2A), where SleB activity in the Δ*cwlJ* mutant causes increased OD loss after 45 min. A potential effect of protease activity on SleB activation during germination might therefore be detectable in a *cwlJ* mutant background. We note a similar difference between the Δ*cwlJ* and the Δ*cwlJ* Δ5 strains, where the Δ*cwlJ* loses OD slightly, but significantly (*P*-value = 0.02), faster than the Δ*cwlJ* Δ5 after 45 min, when SleB exhibits observable activity in this assay (Fig. 2A). The fact that the Δ5 mutant exhibits a greater germination delay than the Δ*sleB* mutant from 5 to 60 min in this assay suggests that the Δ5 mutations might also affect other processes within germination.

### YpeB-Myc is more stable in the absence of the five proteases

**Fig 3:**
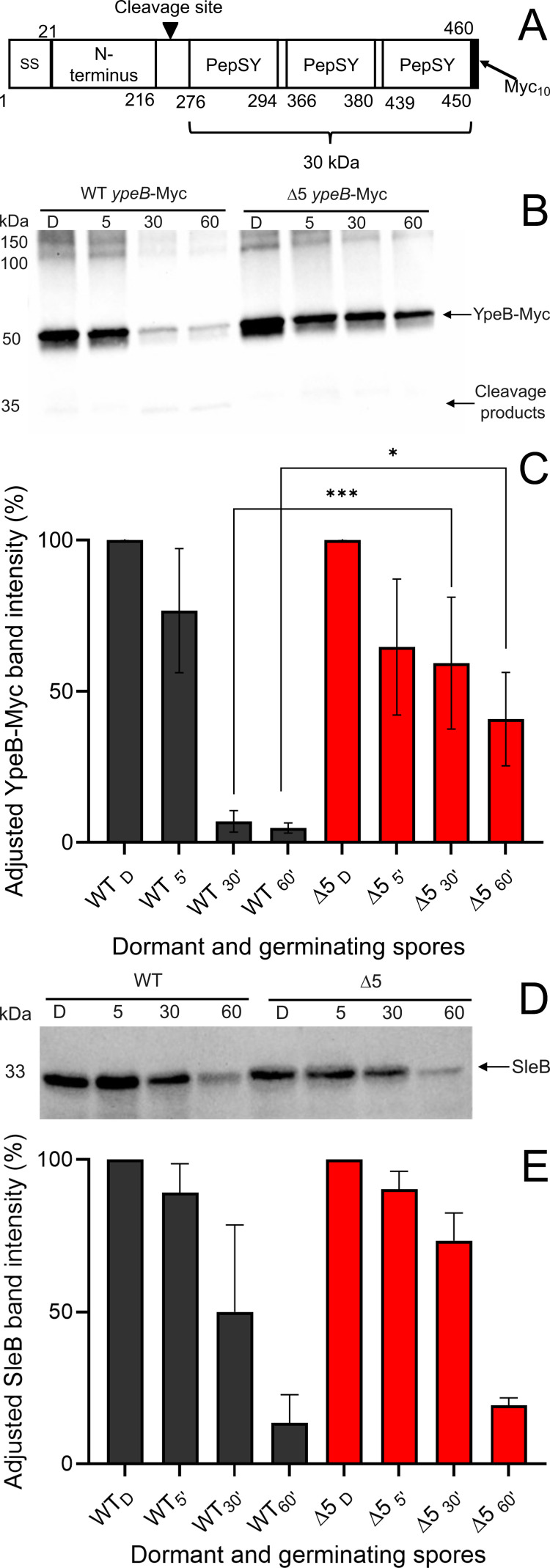
YpeB is more stable in the Δ5 strain during germination of *B. subtilis* spores. (**A**) The scale drawing of the domain architecture of YpeB shows the N-terminal signal sequence (SS), the C-terminal PepSY domains, and the predicted YpeB cleavage sites with the molecular mass of the C-terminal cleavage products. The residue numbers designate the amino acid positions of domain boundaries and the position of YpeB cleavage during spore germination. (**B and D**) Dormant (**D**) spores were germinated with 10 mM L-valine, and samples were collected after 5 (5′), 30 (30′), and 60 (60′) min. Western blot of the extracted proteins probed with anti-Myc (**B**) or anti-SleB (**D**) antibodies as described in Materials and Methods. The strains were DPVB1167 (WT, *ypeB-Myc*) and DPVB1165 (Δ5*, ypeB-Myc*). The positions of the molecular mass marker proteins (not shown) are indicated on the left. (**C and E**) Quantification of the YpeB-Myc and SleB band intensity, respectively, after normalization using a stain-free blot image of the total protein. Plotted points are means ± SD of four biological replicates. *P*-value ≤0.05 (*) and *P*-value ≤0.01 (***) were considered significant.

A previous study indicated that HtrC cleaves YpeB during spore germination in *B. subtilis*; however, a deletion of *htrC* only partially stabilized YpeB during germination (25). YpeB was tagged with Myc at the C-terminus by insertion of an integration vector at the *ypeB* locus. The stability and activity of the YpeB-Myc were tested by Western blot and germination, respectively. Incorporation of SleB into spores is dependent on YpeB (22, 23). YpeB-Myc function can therefore be observed through the amount of SleB in spores and through the activity of SleB in the absence of CwlJ (23, 36, 38). The YpeB-Myc-tagged strain was able to complete germination and form colonies like WT in the absence of CwlJ (Table 3). Anti-SleB Western blot showed a stable SleB in dormant spores (33 kDa) with a decrease in SleB band intensity later during germination (Fig. 3D; Table 3). These data show that the Myc tag does not alter the ability of YpeB to drive SleB incorporation into the spore.

Proteins were extracted from dormant spores and from spores 5, 30, and 60 min after germination initiation. Anti-Myc Western blots revealed that YpeB-Myc is more stable during germination in the absence of these proteases. A 10-fold decrease in YpeB-Myc band intensity was seen after 30 min of germination of the WT but only a 2.7-fold decrease in the Δ5 strain (*P*-value = 0.04). YpeB-Myc is also more stable after 60 min of germination in the absence of the proteases. A ~30 kDa suspected cleavage product, similar to that seen in *B. anthracis* (23), is seen during germination of the WT strain but is undetectable in the Δ5 strain. This is consistent with the data published previously on *B. anthracis* and *B. subtilis* YpeB cleavage in the absence of HtrC (23, 25).

### Delayed SleB function in the absence of the five proteases

**Fig 4:**
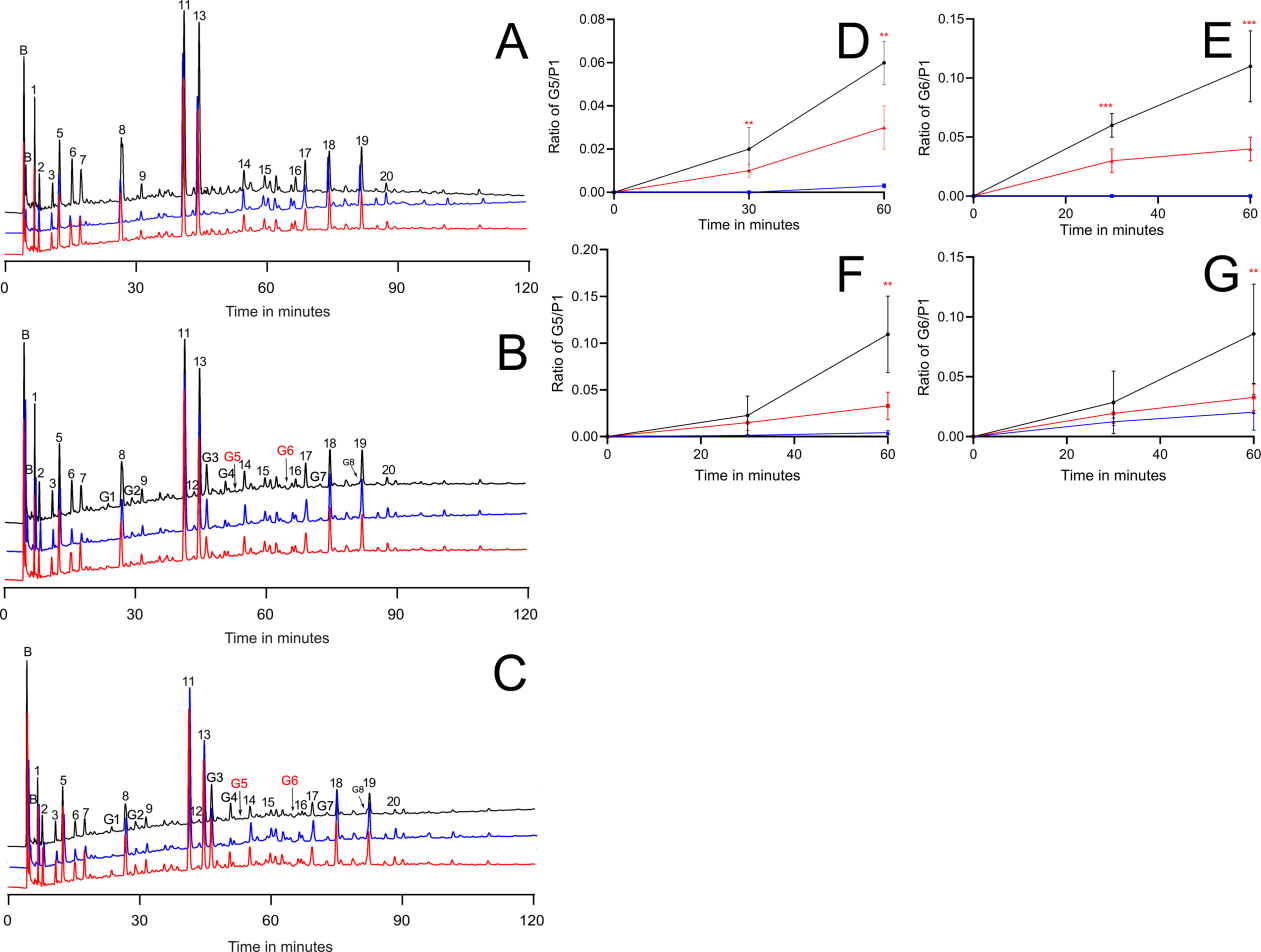
HPLC separation of spore cortex muropeptides. (**A**) Separation of muropeptides produced from dormant WT, Δ*sleB*, and Δ5 mutant spores. (**B**) Separation of muropeptides produced from WT, Δ*sleB*, and Δ*5* mutant spores 30 min and (**C**) 60 min after addition of 10 mM L-Val. Peaks are numbered sequentially according to references 19, 20, 33. B indicates a buffer component. The *x*-axis time scale starts 5 min after sample injection. The *y*-axis is A_206nm_ milliabsorbance units. (**D and E**) Ratios of SleB-dependent germination peaks G5 and G6 to peak 1 in the WT (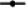), Δ*sleB* (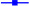), and Δ5 (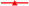) mutant strains. Peak 1 was used for normalization as an internal control for cortex PG recovery and loading. (**F and G**) Total ion counts for muropeptides G5 and G6, normalized to muropeptide 1 as an internal standard, 30 (**F**) and 60 (**G**) min post-germination for the WT (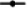), Δ*sleB* (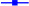), and Δ5 (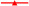). Data were plotted as mean ± SD from four biological replicates for HPLC data (**D and E**) and mean ± SD from three biological replicates for the MS data (**F and G**). *P*-value <0.05 was considered significant (**), and *P*-value <0.01 was considered highly significant (***).

While SleB-dependent muropeptides are detectable in *B. subtilis* spore germination exudate after 120 min (20), we have found that they are barely detectable after 30 and 60 min. We chose to examine SleB activity in the insoluble spore cortex during germination. Cortex peptidoglycan was extracted from DPVB1165 (Δ5, *ypeB-Myc:MLS^r^*), DPVB1167 (*ypeB-Myc:MLS^r^*), and DPVB152 (Δ*sleB*) dormant spores and from germinated spores after 30 and 60 min. Muropeptide samples were analyzed using HPLC (Fig. 4A through C). Peak areas were integrated from the chromatograms, and individual peak areas were compared between dormant spores and germinating spores. Peak area changes from dormant to 30 and 60 min germinated samples were compared (Table S1). Most germination-specific muropeptides were decreased in the Δ5 mutant strain relative to the WT. Germination-specific muropeptides (G5 and G6), which can be attributed directly to SleB activity (20, 36) (Fig. 4B and C; Table 4), were nearly absent in the *sleB* mutant strain (Table S1), and what was detected was likely baseline noise because we chose to integrate some signal in that region of the chromatogram. We examined the ratios of SleB-dependent muropeptide peak areas to the peak area of muropeptide 1, which is thought to be predominantly in the germ cell wall (6) and is therefore not expected to be altered during germination. The ratios of G5 to peak 1 and G6 to peak 1 were decreased in the Δ5 mutant strain at 30- and 60-min post-germination (Table S1; Fig. 4D and E).

**TABLE 4:** Muropeptides identified in the PG of dormant and germinating spores^*a*^

Peak	Peak in (19)	Structure
1	1	DS-TriP
2	2	DS-TriP-gly
3	3	DS-TP
5	5	TS-TP^*b*^^,^^*c*^
6	6	TS-reduced-TP
7	7	TS-reduced-Ala
8	8	DS-TriP-DS-TP
9	9	DS-TP-DS-TP
10	10	TS-TP^*c*^
12	12	DS-TP-TS-reduced-TP
13	13	TS-Ala^*c*^
14	14	DS-TP-TS-TP^*c*^
15	15	HS-reduced-TP
16	16	HS-reduced-TP
17	17	TS-TP-TS-TP^*c*^
18	18	HS-TP^*c*^
19	19	HS-Ala^*c*^
20	20	TS-TP-HS-TP^*c*^
G1	G1	TS-reduced-TP^*d*^
G2	G2	TS-TP^*d*^
G3	G3	TS-TP
G4	G4	TS-Ala^*d*^
G5	G10	AnhydroTS-Ala
G6	G11	AnhydroTS-Ala^*d*^
G7	G6	HS-TP^*d*^
G8	G7	HS-Ala^*d*^

^*a*^ Numbered as indicated in Fig. 4. DS, disaccharide; TS, tetrasaccharide; HS, hexasaccharide; Ala, L-alanine side chain on NAM; TriP, tri-peptide side chain on NAM; TP, tetrapeptide side chain on NAM. Where two saccharide moieties are indicated, they are cross-linked via their peptide side chains.

^*b*^ This molecule contains an unidentified chemical modification (11).

^*c*^ These molecules contain MAL residues (19, 20).

^*d*^ These molecules have a stereochemical alteration (23).

Because peaks G5 and G6 were so small and their quantification was subject to noise in the chromatogram baseline, muropeptides were further analyzed and quantified using LC-MS. The total ion counts of muropeptides 1, G5, and G6 were integrated from the mass spectra. Ion counts were normalized to that of muropeptide 1 as an internal control. The Δ5 strain exhibited a 1.5-fold decrease in the relative abundance of both G5 and G6 when compared to WT 30 min post-germination (Fig. 4F). The relative abundance of G5 in the Δ5 strain exhibited a significant 3.3-fold decrease relative to WT after 60 min of germination, while G6’s relative abundance exhibited a significant 2.6-fold decrease compared to WT (Fig. 4G). G5 and G6 were nearly undetectable in the Δ*sleB* negative control (Fig. 4F and G), and the origins of the weak signals are unknown.

## DISCUSSION

During *B. subtilis* spore germination, YpeB is degraded by HtrC and other proteases, and this may contribute to the onset of cortex degradation by SleB. We have examined the contributions of several spore membrane-associated proteases to spore germination, YpeB degradation, and SleB activity. Germination occurs normally in WT spores and spores lacking one of the six studied proteases (YugP, YtmA, YmfF, YmfH, MlpA, and HtrC) (23). Combined gene deletions of *yugP* and *htrC* cause a small delay in germination; however, adding one or two more deletions of other protease genes did not affect the phenotype of the spores more severely (Fig. S1). Previous studies have shown that MlpA is not essential for the viability, growth, or sporulation of *B. subtilis* (39). This study further demonstrates that the deletion of *mlpA* does not impact spore germination. Combining five protease deletions in one strain, however, resulted in a severe germination defect and caused a delay in OD loss for about 30 min. Δ5 protease mutant spores exhibited normal colony formation efficiency, indicating that the absence of these proteases does not affect the completion of germination and outgrowth of the spores. These proteases could act on a variety of spore components, including the spore coats, either during spore formation or germination, resulting in a germination delay, so we sought more direct evidence that they might exert an effect through YpeB degradation.

YpeB is degraded during the germination of both WT and *htrC* mutant *B. subtilis* spores, as the deletion of HtrC fails to fully stabilize YpeB during this process (25). In contrast, a Δ5 protease-deficient strain further enhances YpeB stability, increasing its levels by 10-fold after 30 min of germination. These data correlate with the delay in OD loss that is seen in the first 30 min of germination of the Δ5 strain. An increase in YpeB stability might explain why the protease-deficient strain exhibits delayed germination, as we hypothesize that the degradation of YpeB helps release SleB to degrade the cortex PG. Stable YpeB cleavage products are detected in germinating WT *B. anthracis* spores (25), while non-specific cleavage products are observed in a Δ*htrC* mutant. Western blotting revealed *B. subtilis* YpeB cleavage products in our study at around 32 kDa in WT spores after 30 min of germination. The cleavage products were undetected in the Δ5 protease-deficient spores, further validating the increased stability of YpeB during germination of this strain. The failure to fully stabilize YpeB in the absence of five proteases indicates that either an additional protease(s) is present or that YpeB is slowly released from the membrane and from the spore as germination progresses.

SleB function was studied using HPLC separation of cortex muropeptides. SleB-dependent muropeptides G5 and G6 (19, 40) were absent in germinating Δ*sleB* spores. These muropeptides had a decreased abundance 30 and 60 min post-germination of the Δ5 strain when compared to the WT. Further quantification of the SleB-dependent G5 peak using LC-MS showed a significant decrease in G5 abundance when compared to WT 30 and 60 min post-germination. A larger decrease was observed in G6 relative abundance in the Δ5 strain at both 30 and 60 min post-germination. The decreased abundance of G5 and G6 indicates a decrease in the SleB activity in the protease mutant strain. A delay in SleB activity might explain the defect in germination, as *sleB*-deficient spores exhibit a somewhat less effective germination response, along with a slower rate of outgrowth (36). The germination kinetics of the Δ5 protease-deficient spores was more delayed compared to the *sleB*-deficient spores, further suggesting that the protease deletions might affect SleB activity through delaying YpeB degradation but also through other unknown mechanisms. Altered *B. anthracis* YpeB proteolysis by mutating the cleavage sites targeted by HtrC, however, did not slow spore germination and outgrowth (25). This could be explained by the fact that YpeB was still degraded by other proteases (25).

**Fig 5:**
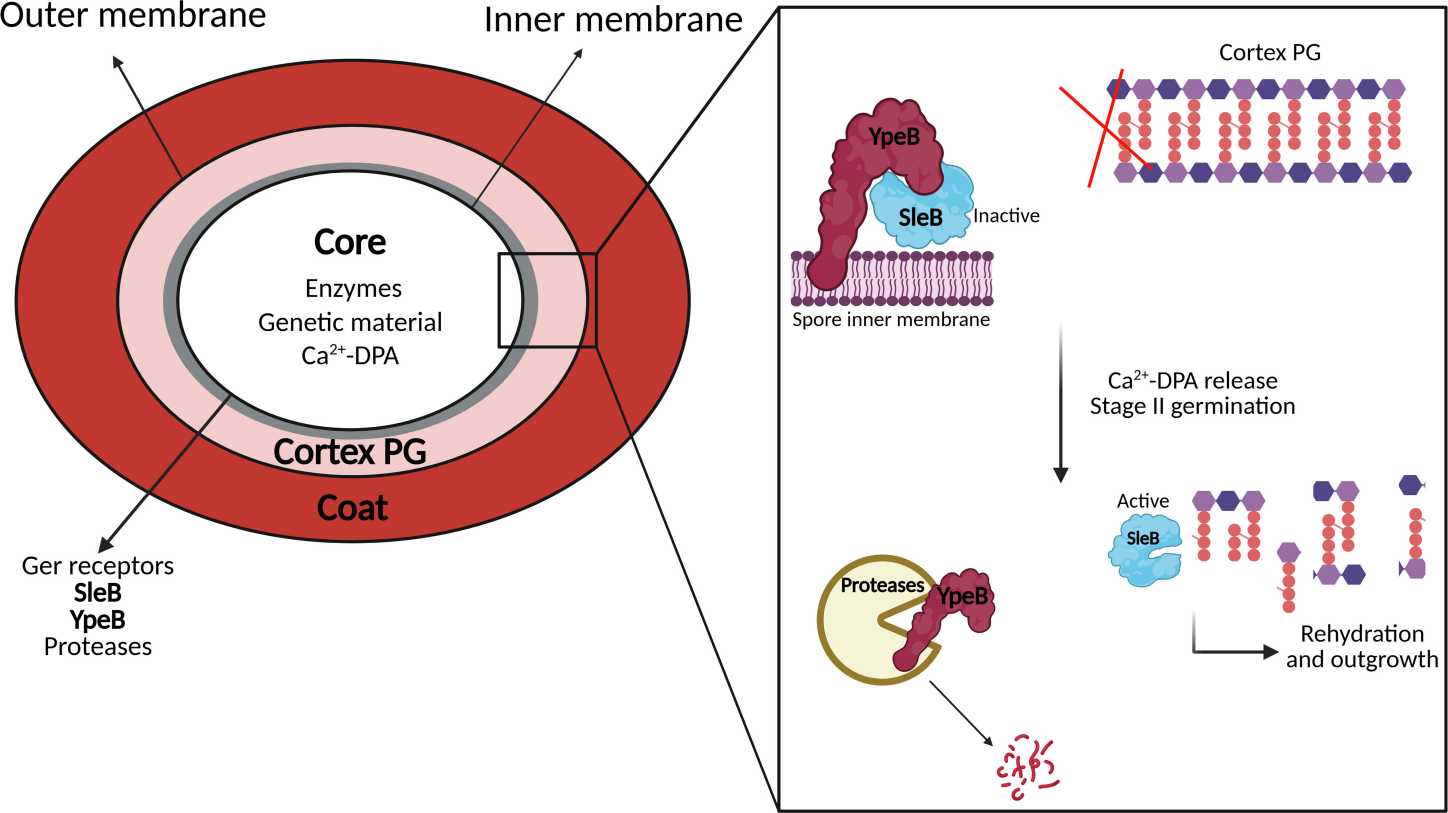
Working model on the activation of SleB after YpeB degradation during spore germination. Both SleB and YpeB are stable on the inner spore membrane in dormant spores, as illustrated in the zoomed-in image. Since YpeB is anchored to the membrane, it is hypothesized that SleB is held inactive by YpeB on the inner spore membrane. Several proteases are involved in YpeB degradation, and this might relieve SleB from inhibition, allowing for cortex degradation and germination completion.

Germination kinetics are altered in *B. subtilis* spores lacking five proteases. It has been previously shown that protease inhibitors block spore germination at various stages, indicating that protease activity plays a crucial role during germination (41, 42). It has not been proven that this inhibition is specifically due to protease blockage rather than interference with another protein function or which exact germination processes are impacted. This study and our findings suggest that protease activity may speed the initiation of cortex degradation by SleB. Figure 5 presents a model in which SleB is held inactive in a complex on the outer surface of the dormant spore membrane. A published model of a YpeB-SleB complex suggests that the SleB active site would be oriented toward the membrane and away from the cortex (43). Appearance of protease activity early during germination could cleave YpeB, releasing SleB to act on the cortex. Proteases associated with this membrane environment could potentially become active during germination due to an increase in membrane fluidity or the release of spore core solutes (10). Previously identified sites at which HtrC cleaves YpeB (25) are exposed in the predicted YpeB-SleB model (43).

The clostridial GSLE SleC must be proteolytically processed to become active, further validating the importance of proteases in spore germination (44, 45). Inhibiting proteases might increase YpeB stability during germination, which in turn can delay SleB activity and alter germination. Alternatively, increasing protease activity during germination might increase germination efficiency, rendering spores more susceptible to killing. More research into how proteases work during spore germination and whether they directly influence cortex degradation could lead to better methods for spore decontamination.

## SUPPLEMENTAL MATERIAL

Table S1 and Figure S1 jb.00145-26-s0001.pdf Table S1: Peak area analyses of muropeptides produced during spore germination. Figure S1: Assay of spore germination for mutants lacking multiple proteases.

ASM does not own the copyrights to Supplemental Material that may be linked to, or accessed through, an article. The authors have granted ASM a non-exclusive, world-wide license to publish the Supplemental Material files. Please contact the corresponding author directly for reuse.
