# Supplementary material for: YpeB stability affects germination possibly through delaying SleB activity in Bacillus subtilis
Source: J Bacteriol. 2026 Jun 22;208(7):e00145-26. doi: 10.1128/jb.00145-26 (PMC13393411; doi:10.1128/jb.00145-26)
Supplement: Table S1 and Figure S1 — Table S1: Peak area analyses of muropeptides produced during spore germination. Figure S1: Assay of spore germination for mutants lacking multiple proteases. [file jb.00145-26-s0001.pdf]

Supplementary Table 1. Muropeptide peak area and the ratio of G5/P1 and G6/P1 during germination.

Replicate #	WT					Δ*sleB*					Δ5				
	Dormant					Dormant					Dormant				
	P1	G5	G6	G5/P1	G6/P1	P1	G5	G6	G5/P1	G6/P1	P1	G5	G6	G5/P1	G6/P1
1	1601	5	7	0.00	0.00	1510	1	3	0.00	0.00	1338	7	8	0.01	0.01
2	1877	8	6	0.00	0.00	1222	3	4	0.00	0.00	1780	5	2	0.00	0.00
3	2657	2	3	0.00	0.00	2063	4	5	0.00	0.00	2622	4	6	0.00	0.00
4	2534	3	5	0.00	0.00	2145	3	3	0.00	0.00	2945	5	4	0.00	0.00
	30’					30’					30’				
1	1285	30	99	0.02	0.08	899	3	4	0.00	0.00	1087	20	40	0.02	0.04
2	982	12	50	0.01	0.05	1027	9	9	0.01	0.01	1213	10	35	0.01	0.03
3	2105	45	131	0.02	0.05	2066	4.5	4	0.00	0.00	2177	25	54	0.01	0.02
4	2795	50	140	0.01	0.05	2294	3	5	0.00	0.00	2631	45	53	0.02	0.02
	60’					60’					60’				
1	941	60	140	0.06	0.15	1130	5	5	0.00	0.00	922	45	52	0.05	0.06
2	748	57	80	0.08	0.11	893	10	9	0.01	0.01	1031	40	45	0.04	0.04
3	1576	80	160	0.05	0.10	2021	5	5	0.00	0.00	1996	40	55	0.02	0.03
4	2183	100	200	0.05	0.09	1927	4	5	0.00	0.00	2135	60	58	0.03	0.03

The peak areas of P1, G5 and G6 were extracted from the chromatograms and the ratios of G5/P1 and G6/P1 were calculated. Data are from four biological replicates.

A

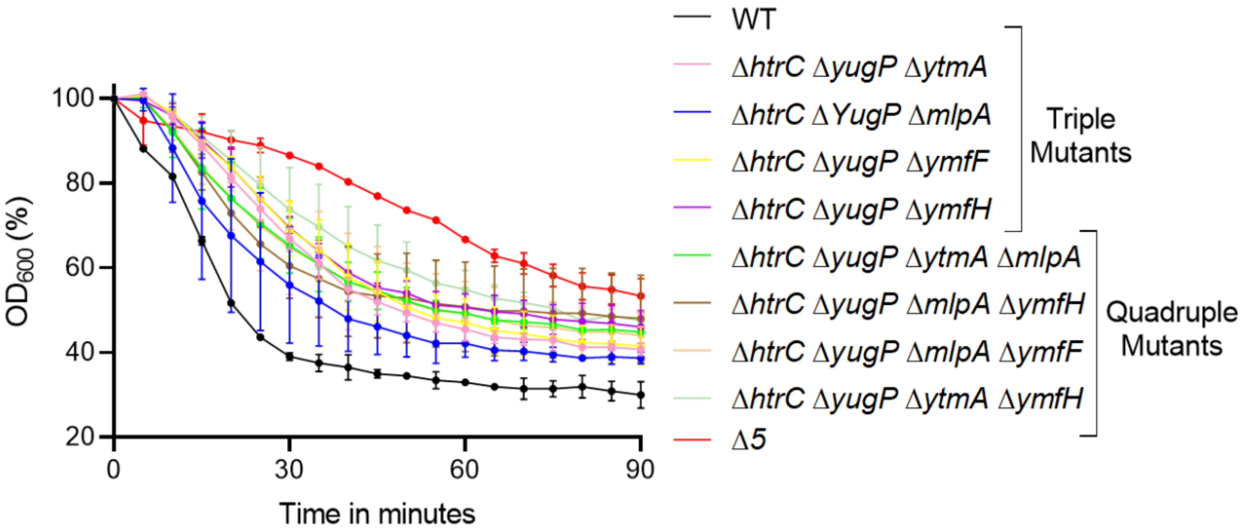

B

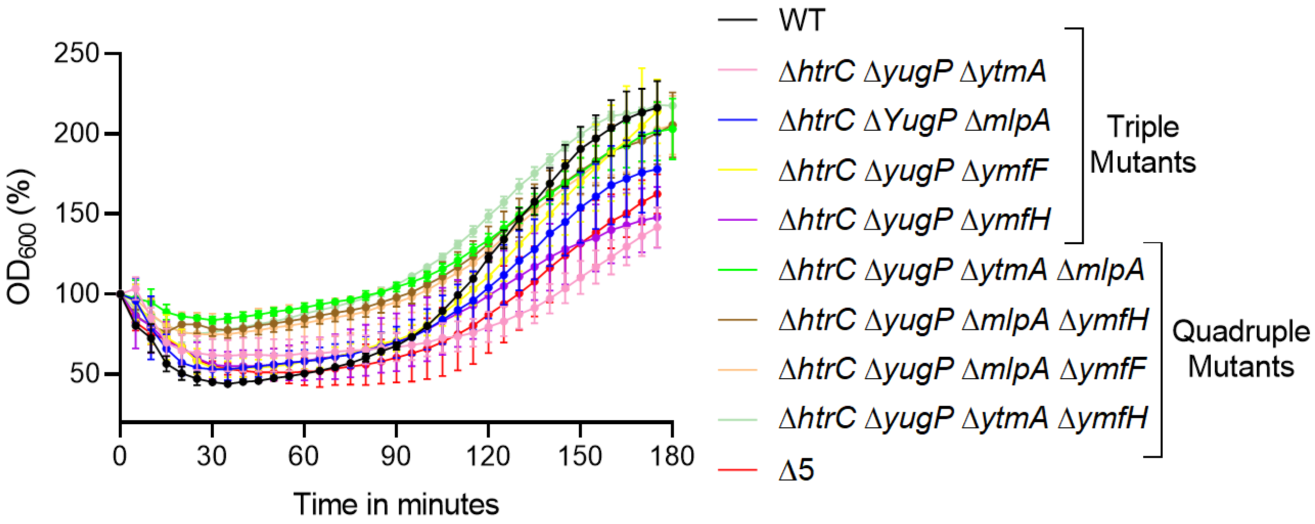

Supplementary Figure 1. Effects of protease-encoding gene deletions on rates of germination and outgrowth of *B. subtilis* spores. Spores were prepared and purified as described in Materials and Methods. For germination (A), spores were heat-activated, chilled, and exposed to 10 mM L-Valine, and OD was tracked and plotted as a percentage of initial OD. For outgrowth (B), spores were germinated in 2xYT. Plotted points are means ±SD of five biological replicates.
